# Current Immunotherapeutic Strategies Targeting the PD-1/PD-L1 Axis in Non-Small Cell Lung Cancer with Oncogenic Driver Mutations

**DOI:** 10.3390/ijms23010245

**Published:** 2021-12-27

**Authors:** Ichidai Tanaka, Masahiro Morise

**Affiliations:** Department of Respiratory Medicine, Nagoya University Graduate School of Medicine, Nagoya 466-8550, Japan; morisem@med.nagoya-u.ac.jp

**Keywords:** immune-checkpoint blockade, PD-1, PD-L1, NSCLC, driver mutation

## Abstract

Treatment strategies targeting programed cell death 1 (PD-1) or its ligand, PD-L1, have been developed as immunotherapy against tumor progression for various cancer types including non-small cell lung cancer (NSCLC). The recent pivotal clinical trials of immune-checkpoint inhibiters (ICIs) combined with cytotoxic chemotherapy have reshaped therapeutic strategies and established various first-line standard treatments. The therapeutic effects of ICIs in these clinical trials were analyzed according to PD-L1 tumor proportion scores or tumor mutational burden; however, these indicators are insufficient to predict the clinical outcome. Consequently, molecular biological approaches, including multi-omics analyses, have addressed other mechanisms of cancer immune escape and have revealed an association of NSCLC containing specific driver mutations with distinct immune phenotypes. NSCLC has been characterized by driver mutation-defined molecular subsets and the effect of driver mutations on the regulatory mechanism of PD-L1 expression on the tumor itself. In this review, we summarize the results of recent clinical trials of ICIs in advanced NSCLC and the association between driver alterations and distinct immune phenotypes. We further discuss the current clinical issues with a future perspective for the role of precision medicine in NSCLC.

## 1. Introduction

Programed cell death 1 (PD-1), a member of the CD28 family, is a key immune-checkpoint receptor that is primarily expressed on a variety of immune cells, including activated T, B, regulatory T (Treg), dendritic (DCs), and natural killer cells [1,2]. The main ligand of PD-1, programed death 1 ligand-1 (PD-L1), also known as B7 homolog 1 or CD274, is constitutively expressed or upregulated in various types of tumor cells. The PD-1/PD-L1 axis inhibits T lymphocyte proliferation, cytokine production, and cytolytic activity, and is pivotal for innate or adaptive immune resistance during tumor development. Monoclonal antibodies that block this interaction can reactivate T-cell activity against tumor cells. Therefore, treatment strategies targeting PD-1 or PD-L1 have been developed as immunotherapy against tumor progression over the past decade [3]. These include the use of immune-checkpoint inhibitors (ICIs), such as anti-PD-1 and anti-PD-L1 antibodies, which result in prolonged survival in patients with the various types of cancer, including non-small cell lung cancer (NSCLC), which is the most prevalent form of lung cancer.

NSCLC has been characterized by driver mutation-defined molecular subsets and various molecular targeted therapies against NSCLC with oncogenic driver mutations have been developed that dramatically improve patient outcome. Meanwhile, prior to the establishment of immune therapies with ICIs, the therapy for advanced NSCLC without targetable driver alterations included platinum-based cytotoxic chemotherapy and the overall three-year survival rate of stage IV NSCLC was less than 10% [4,5]. However, the clinical trials of ICIs combined with chemotherapy have reshaped NSCLC therapy and to yield a variety of first-line treatment options for patients. The growing use of ICIs has dramatically improved patient outcome and the overall three-year survival rate of stage IV NSCLC has reached 40–50% [6,7,8,9,10,11,12,13]. The therapeutic effects of ICIs in these clinical trials have been associated with PD-L1 tumor proportion scores (TPS) or tumor mutational burden (TMB). In general, PD-1 or PD-L1 inhibitors show excellent therapeutic effects against tumors that express high PD-L1 levels (TPS > 50%) and/or a high TMB [6,7,8,9,10,11,12,13,14]; however, these indicators are not sufficient to predict response or outcome to ICI therapy [15,16,17,18]. Consequently, other biological approaches have been used to identify predictive biomarkers and identify mechanisms of cancer immune escape. Multi-omics analyses, including next generation sequencing (NGS)-based tests, have revealed that NSCLC with specific driver mutations exhibit discrete immune phenotypes [19,20,21,22]. Of these, co-occurring *Kirsten rat sarcoma viral oncogene homolog* (*KRAS*) mutation and tumor protein P53 (TP53) inactivation are associated with T-cell infiltration and reflect a favorable response to ICIs with high expression of PD-L1 and other tumor antigens [23,24]. Conversely, two tumor suppressor genes, liver kinase B1 (LKB1) and kelch-like ECH-associated protein 1 (KEAP1), represent inactivating mutations associated with an immunosuppressed phenotype [19]. These driver mutations affect the regulatory mechanism of PD-L1 expression on the tumor itself and such tumors are resistant to ICIs regardless of PD-L1 expression and high TMB. Currently, NGS-based assays are beginning to decline in cost and will become more widely utilized to identify driver mutations, which will help to identify ICI-responding patients.

Because NSCLC is a heterogeneous disease, a complete understanding of the association between PD-L1 expression and driver gene alterations will guide ICI utilization. In this review, we summarize pivotal clinical trials of ICIs in advanced NSCLC, the therapeutic efficacy of PD-1 or PD-L1 inhibitors, and the mechanisms of PD-L1 upregulation in the context of intrinsic pathways that rely upon specific driver alterations. Moreover, we discuss current clinical issues and future perspective for precision medicine in NSCLC.

## 2. Current ICI Therapeutic Strategies in NSCLC

### 2.1. Mono Therapy with PD-1/PD-L1 Inhibitors

Based on the early development of PD-1/PD-L1 inhibitors in NSCLC, several important phase III studies were conducted. The results showed a clear survival benefit of PD-1/PD-L1 inhibitors compared with cytotoxic chemotherapy, which had been the standard treatment for decades. First, PD-1/PD-L1 inhibitors were compared with docetaxel as that was a standard agent used in a pretreatment setting. Nivolumab, pembrolizumab, and atezolizumab exhibited superior survival in clinical studies. CheckMate 017 (nivolumab for squamous NSCLC) [25], CheckMate 057 (nivolumab for non-squamous NSCLC) [26], and OAK (atezolizumab for NSCLC) [27] enrolled patients regardless of PD-L1 expression status. In contrast, Keynote-010 (pembrolizumab for NSCLC) enrolled patients with only a PD-L1 TPS > 1% [28]. These trials all demonstrated an overall survival (OS) benefit for PD-1/PD-L1 inhibitors with an HR of 0.59–0.73 compared with docetaxel. Based on the initial positive results PD-1/PD-L1 for inhibitors, clinical development shifted to patients with untreated NSCLC as shown in Table 1. Because PD-1/PD-L1 inhibitors exhibited a higher tumor response, especially in patients with positive PD-L1 expression in tumors compared with negative PD-L1 expression, further phase III studies including Checkmate026, KEYNOTE-024, KEYNOTE-042, and IMpower110 were conducted to compare PD-1/PD-L1 inhibitors to platinum-doublet therapy in a front-line setting focused on patients with positive PD-L1 expression [29,30,31,32]. The companion diagnostic test for PD-L1 expression was different from each other and patient enrollment in each phase III study was selected according to each companion diagnostic test. Of these studies, KEYNOTE-024, KEYNOTE-042, and IMpower 110 demonstrated a survival benefit of front-line PD-1/PD-L1 inhibitors compared with platinum-based chemotherapy [30,31,32]. In patients with strong positive PD-L1 expression (PD-L1 TPS ≥ 50% for pembrolizumab and TC3 or IC3 for atezolizumab), PD-1/PD-L1 inhibitors demonstrated a definite survival benefit compared with platinum-based chemotherapy. However, in patients with weakly-positive PD-L1 expression (PD-L1 TPS 1–49% for pembrolizumab and TC1/2 or IC1/2 for atezolizumab), the superiority of PD-1/PD-L1 inhibitors to platinum-based chemotherapy was not as clear according to a subgroup analysis of these phase III studies. Collectively, pembrolizumab and atezolizumab are regarded as standard treatment options for patients exhibiting strong positive tumor PD-L1 expression.

### 2.2. PD-1/PD-L1 Inhibitors in Combination with Platinum-Based Chemotherapy

In parallel with the clinical development of front-line PD-1/PD-L1 inhibitors, phase III trials with or without PD-1/PD-L1 inhibitors in combination with platinum-based chemotherapy were conducted in the last five years. These studies targeted chemotherapy naïve patients regardless of PD-L1 expression status. For non-squamous NSCLC, four phase III trials showed a clinical benefit of PD-1/PD-L1 inhibitors in combination with platinum-based chemotherapy. Carboplatin (CBDCA)/Pemetrexed (PEM)/Pembrolizumab, CBDCA or CDDP/PEM/atezolizumab, CBDCA/nanoparticle albumin bound-Paclitaxel (nabPTX)/atezolizumab, CBDCA/PTX/bevacizumab (BEV)/atezolizumab and CBDCA/PTX/BEV/nivolumab are now standard treatment options for non-squamous NSCLC in a front-line setting [6,7,8,9,10]. With respect to squamous NSCLC, phase III trials comparing CBDCA/PTX or nabPTX/pembrolizumab to CBDCA/PTX or nabPTX showed a survival benefit [33]. These trials provided multiple treatment options for PD-1/PD-L1 inhibitors in combination with platinum-based chemotherapy.

PD-1/PD-L1 inhibitors in pretreated NSCLC showed a poor clinical benefit for patients with *Epidermal Growth Factor Receptor* (*EGFR*) mutation or *Anaplastic Lymphoma Kinase* (*ALK*) gene fusion. This affected patient selection for front-line phase III studies of PD-1/PD-L1 inhibitors in combination with platinum-based chemotherapy. All studies described above enrolled patients without *EGFR* mutation or *ALK* fusion as a study population. IMpower150 initially included patients with *EGFR* mutation or *ALK* fusion, however, the primary analysis population was amended to exclude patients with *EGFR* mutation or *ALK* fusion [9]. Therefore, we only have the results of exploratory data analysis for CBDCA/PTX/BEV/atezolizumab in patients with *EGFR* mutation or *ALK* fusion. The HR of CBDCA/PTX/BEV/atezolizumab to CBDCA/PTX/BEV in patients with sensitizing *EGFR* mutations was 0.31 (95%CI, 0.11–0.83) [11]. The data suggests that the addition of BEV resulted in a clinical benefit for PD-1/PD-L1 inhibitors in combination with platinum-based chemotherapy for patients with a sensitizing *EGFR* mutation. Indeed, NSCLC patients with a sensitizing *EGFR* mutation exhibited higher *VEGF-A* expression in tumors compared with those containing wild-type EGFR, and the benefits of adding BEV was better in patients with a sensitizing *EGFR* mutation compared with the wild-type EGFR NSCLC patients [34]. Further studies of PD-1/PD-L1 inhibitors in combination with platinum-based chemotherapy for patients with *EGFR* mutation or *ALK* fusion are warranted.

### 2.3. PD-1/PD-L1 Inhibitors in Combination with Cytotoxic T-Lymphocyte-Associated Protein 4 (CTLA-4) Inhibitors

Cytotoxic T-lymphocyte antigen 4 (CTLA-4) is a member of the CD28 receptor family and counteracts the interaction between CD28 on the surface of naïve T cells and B7 ligands on the surface of antigen-presenting cells (APCs), which is an important role for T-cell activation as a costimulatory pathway [35,36]. CTLA-4 is constitutively highly expressed on regulatory T cells (Treg) and is induced in activated T cells after the T-cell receptors bind to antigens presented by APCs in conjunction with the CD28-B7 interaction [37]. Because CTLA4 has a much higher affinity for B7 ligands than CD28, it can interfere with the CD28-B7 interaction resulting in the suppression of T-cell activation and proliferation [38,39]. The anti-CTLA4 antibody, ipilimumab, binds to CTLA4 on Tregs and blocks the counter-function to the CD28-B7 costimulatory pathway. Simultaneously, ipilimumab also blocks CTLA4 on the surface of conventional T cells and promotes T-cell activation. Furthermore, ipilimumab can bind to the Fc receptor on APCs and induce antibody-dependent cellular cytotoxicity to Tregs. Thus, ipilimumab enhances the T-cell clonal response to tumor neoantigens and shows a favorable response to tumors with high TMB [40,41,42].

CTLA4 regulates T cells differently than the PD-1/PD-L1 axis and the combination of ipilimumab with anti-PD1 or anti-PD-L1 antibodies demonstrated synergic effects in suppressing the development of various tumors, such as melanoma and NSCLC [43,44]. The clinical benefits of nivolumab/ipilimumab in NSCLC were investigated in the CheckMate 227 trial [45,46]. The study design included PD-1/PD-L1 inhibitors in combination with platinum-based chemotherapy, whereas platinum-based chemotherapy was used as a control arm. CheckMate 227 enrolled both patients with a PD-L1 expression level of 1% or more and those with a PD-L1 expression level of less than 1% separately. One of the primary endpoints was OS in patients with a PD-L1 expression level of 1% or more in the trial. Exploratory analysis comparing nivolumab/ipilimumab to platinum-based chemotherapy was also conducted in patients with a PD-L1 expression level of less than 1%. Nivolumab/ipilimumab demonstrated a survival benefit compared with platinum-based chemotherapy in the PD-L1-positive population. Although it was just exploratory, nivolumab/ipilimumab was also superior to platinum-based chemotherapy in PD-L1 negative patients. CheckMate 227 was the first pivotal trial that demonstrated the benefit of adding a PD-1 inhibitor plus a CTLA4 inhibitor to platinum-based chemotherapy. This raised the question as to whether the addition of a CTLA4 inhibitor to a PD-1 inhibitor was beneficial in patients with strong positive PD-L1 expression. Recently, the results of KEYNOTE-598 comparing pembrolizumab plus ipilimumab to pembrolizumab in patients with a PD-L1 TPS ≥ 50% were reported [12]. The study concluded that adding ipilimumab to pembrolizumab did not improve efficacy compared with pembrolizumab monotherapy as first-line treatment. There is no direct comparison data between nivolumab/ipilimumab and pembrolizumab in patients with PD-L1 TPS ≥ 50%. However, long term follow-up data for pembrolizumab or atezolizumab monotherapy has revealed a survival benefit compared with platinum-based chemotherapy in patients with strong, positive PD-L1 expression. Further clinical development in this population will require randomized trials that set pembrolizumab or atezolizumab monotherapy, but not platinum-based chemotherapy, as the control arm.

### 2.4. PD-1/PD-L1 Inhibitors Plus CTLA-4 Inhibitors in Combination with Platinum-Based Chemotherapy

In recent years, new evidence of ICI combination has emerged. CheckMate 9LA study compared nivolumab plus ipilimumab (CTLA-4 inhibitor) combination with platinum-based chemotherapy to platinum-based chemotherapy in patients with NSCLC regardless PD-L1 expression [13]. In the study, CBDCA/PEM/nivolumab/ipilimumab for non-squamous NSCLC and CBDCA/nabPTX or PTX/nivolumab/ipilimumab for squamous NSCLC was selected as experimental arm. These quadruple regimens showed survival benefit compared to platinum-based chemotherapy in intent to treat population, thus we can now choice the quadruple regimens instead of PD-1/PD-L1 inhibitors combination with platinum-based chemotherapy for both non-squamous NSCLC and squamous NSCLC. Further, another data of PD-1/PD-L1 inhibitors plus CTLA-4 inhibitors combination with platinum-based chemotherapy were published. Durvalumab plus tremelimumab combination with platinum-based chemotherapy was compared to platinum-based chemotherapy in POSEIDON. Consistent with the results of 9LA, durvalumab plus tremelimumab with combination with platinum-based chemotherapy showed positive results [47].

## 3. The Association between PD-L1 Expression and Efficacy of ICIs in NSCLC with Driver Gene Alterations

### 3.1. PD-L1 Upregulation Mechanisms in NSCLC with KRAS Mutation

**Figure 1:**
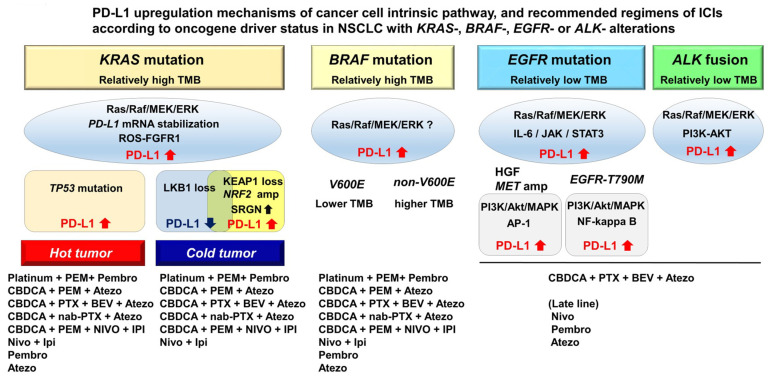
Mechanisms of PD-L1 upregulation of cancer cell intrinsic pathways and recommended ICI regimens based on oncogene driver status in NSCLC with *KRAS*-, *BRAF*-, *EGFR*-, or *ALK*- alterations.

Keeping pace with the progression of ICI development, various regulatory mechanisms of PD-L1 expression have been studied and are known to be affected by a variety of factors including inflammatory signals, mechanical signals, and intrinsic cell signaling [48,49,50,51]. The representative tumor-intrinsic signals include AKT serine/threonine kinase 1 (AKT)-mammalian target of rapamycin signaling, MYC protooncogene, Signal transducer and activator of transcription 3 (STAT3), and nuclear factor kappa-light-chain-enhancer of activated B cells (NF-κB), which have been observed in various cancer types [51]. Among the investigations of PD-L1 regulation in NSCLC with *KRAS* mutation, Chen et al. found that upregulation of *PD-L1* mRNA expression induced by KRAS activation resulted from phosphorylation of extracellular signal-regulated kinase (ERK), but not phosphorylation of AKT [52]. Subsequently, Coelho et al. discovered that PD-L1 expression in tumor cells may be driven by the Ras/Raf/Mitogen-activated protein kinase dB (MEK)/ERK pathway through stabilization of *PD-L1* mRNA, based on modulation of the AU-rich elements in the 3′-untranslated region (UTR) [53]. This pathway phosphorylates and inhibits the AU-rich element-binding protein, tristetraprolin (TTP), that negatively regulates *PD-L1* expression. This molecular mechanism is consistent with the invariable stabilization of aberrant *PD-L1* transcripts by structural variations, which disrupt the 3′-region of the *PD-L1* gene through 3′-UTR truncation in multiple cancer types, such as adult T-cell leukemia/lymphoma and diffuse large B-cell lymphoma [54]. Furthermore, a recent study demonstrated that activation of oncogenic KRAS also enhanced PD-L1 expression through a redox-mediated mechanism. Oncogenic KRAS signaling accelerates the generation of reactive oxygen species and induces expression of fibroblast growth factor receptor 1 (FGFR1), resulting in increased PD-L1 expression [55]. These studies indicate that multiple molecular mechanisms can be cooperative to increase PD-L1 expression in NSCLC with *KRAS* mutation (Figure 1).

Non-small cell lung cancer (NSCLC) with specific driver alterations exhibits a discrete immune phenotype. NSCLC with *Kirsten rat sarcoma viral oncogene homolog (KRAS)* or *B-Raf Proto-Oncogene (BRAF)* mutations have a higher TMB, whereas NSCLC with *EGFR mutation* or *ALK fusion* exhibits a lower TMB, resulting in an unfavorable response to monotherapy with PD-1/PD-L1 inhibitors. Depending on these driver alterations, various intrinsic pathways are involved in PD-L1 regulation. Co-occurring *KRAS* mutations and TP53 inactivation are associated with T-cell infiltration and reflect favorable responses to monotherapy with PD1/PD-L1 inhibitors with high PD-L1 expression. Conversely, *KRAS* mutation and inactivating mutations of *liver kinase B1* (*LKB1*) or *kelch-like ECH-associated protein 1* (*KEAP1*), or persistent activation of *nuclear factor erythroid 2-related factor 2* (*NRF2*) are associated with immunosuppressed phenotypes. NSCLC with *LKB1* inactivation or disruption of the KEAP1-NRF2 pathway exhibits primary resistance to PD-1/PD-L1 blockade of PD-L1 expression and high TMB.

CBDCA, carboplatin; PEM, pemetrexed; nab-PTX, nanoparticle albumin bound-Paclitaxel; PTX, Paclitaxel; BEV, bevacizumab; Nivo, nivolumab; Ipi, ipilimumab; Pembro, pembrolizumab; Atezo, atezolizumab.

### 3.2. Heterogeneity of PD-L1 Expression and ICIs Efficacy in NSCLC with KRAS Mutation

Positive PD-L1 staining was more frequent in patients with *KRAS* mutation compared with wild-type *KRAS* patients in the KEYNOTE-001 study [56]. Consistently, monotherapy with anti-PD-1 antibodies, such as nivolumab or pembrolizumab, initially showed a greater clinical benefit in patients with *KRAS* mutation compared with *KRAS* wild-type patients [56]. However, a multi-omics analysis uncovered the heterogeneity of *KRAS*-mutant lung adenocarcinomas based on co-occurring genetic alterations including inactivation of TP53 or LKB1 and low expression of the thyroid transcription factor-1 (TTF-1) [23,24]. The integrative analysis with clinical data indicated that these distinct subsets affect PD-L1 expression and the response to PD-1/PD-L1 inhibitors (Figure 1). Among them, *KRAS*-mutant lung adenocarcinomas with TP53 inactivation is characterized as high PD-L1 expression together with high TMB and marked T-cell infiltration, and showing favorable responses to monotherapy with anti-PD-1/PD-L1 antibodies [23,24].

In contrast to TP53 inactivation, lung adenocarcinomas with LKB1 inactivation, which is encoded by *serine/threonine kinase 11*, is associated with the downregulation of PD-L1 expression and reduced T-cell infiltration. Somatic mutation of *LKB1* occurs in approximately 20% of lung adenocarcinomas and 30% of *KRAS*-mutant lung adenocarcinomas, whereas LKB1 inactivation is present as a germline mutation of the autosomal dominant disorder, Peutz–Jeghers syndrome [23,24,57]. The loss of LKB1 function affects tumor initiation though the dysregulation of cell polarity and the reprograming of energy metabolism, including glucose uptake and pyrimidine/purine balance [58,59,60,61]. These drastic intracellular transformations can affect the secretion of proinflammatory cytokines, such as interleukin-6 (IL-6) and chemokine (C-X-C motif) ligand 7 (CXCL7), resulting in the accumulation of immunosuppressive neutrophils and exhausted or suppressed infiltrated T cells [62]. Consistent with these basic molecular analyses, a pan-cancer T-cell-inflamed gene expression profile (GEP) consisting of 18 genes, which represent the T-cell-activated tumor microenvironment (TME), revealed that somatic mutation of LKB1 was one of the most prevalent driver alterations in immunosuppressed phenotypes in NSCLC known as “cold tumor” [19]. In fact, anti-PD-1/PD-L1 monotherapy is ineffective in NSCLC with LKB1 inactivation, which exhibits primary resistance to PD-1/PD-L1 blockade with PD-L1 negativity and intermediate or high TMB [63].

The *KEAP1* inactivating mutation is associated with the immunosuppressive phenotype and is frequently involved in TTF-1-negative lung adenocarcinoma, which was reported as deficient T-cell infiltration from the analysis of a pan-cancer T-cell-inflamed GEP [64]. KEAP1 is a redox-regulated substrate for the cullin-3 dependent E3 ubiquitin ligase complex, which facilitates the ubiquitination and subsequent proteolysis of nuclear factor erythroid 2-related factor 2 (NRF2), a master regulator of detoxification, antioxidant response, and anti-inflammatory activity [65]. KEAP1 inactivation results in persistent NRF2 activation; therefore, the tumors are highly resistant to radiotherapy and cytotoxic chemotherapy [65,66,67]. KEAP1 inactivation is also involved in reprograming to an immunosuppressive TME through Srglycin (SRGN) secretion, which is a chondroitin sulfate proteoglycan that plays an intricate role in inflammation by regulating several inflammatory mediators [68,69,70]. SRGN expression is transcriptionally upregulated by NRF2 activation and epigenetically induced through nicotinamide N-methyltransferase-induced perturbation of methionine metabolism in TTF-1–negative lung adenocarcinoma [70]. Cancer cell-derived SRGN upregulates PD-L1 expression on the cancer cell itself and increases the secretion of proinflammatory cytokines, including IL-6, interleukin-8 (IL-8), and chemokine (C-X-C motif) ligand 1 (CXCL1), indicating that it contributes to reprogramming into an aggressive and immunosuppressed phenotype [70,71]. Similar to NSCLC with LKB1 inactivation, NSCLC with disruption of the KEAP1-NRF2 pathway is widely known respond poorly to monotherapy with anti-PD-1/PD-L1 antibodies. Arbour et al. analyzed co-occurring the genetic alterations of 330 patients with *KRAS*-mutant NSCLC by NGS and found that *KEAP1-NRF2* alterations occurred in 27% of the patients that had shorter OS from the initiation of immunotherapy [23]. Furthermore, a subset of NSCLC harbors inactivating mutations of both *LKB1* and *KEAP1/NRF2* and demonstrate a further aggressive clinical course with strong resistance to ICIs treatment [72]. Papillon-Cavanagh et al. analyzed the clinical efficacy of PD1/PD-L1 inhibitors or platinum-based chemotherapy against NSCLC with the double-mutational status in a real world-setting. Patient outcome for both treatments was worse progression-free survival (PFS) and OS compared with patients harboring only an *LKB1* alteration, only *KEAP1/NRF2* alterations, or a negative status for both [73]. These results indicate that co-occurring genetic alterations of *LKB1* and *KEAP1/NRF2* have an additive effect for tumor aggressiveness even with combined ICI regimens containing cytotoxic chemotherapy. To improve the outcome of NSCLC with these aggressive phenotypes, new therapeutic developments are needed.

### 3.3. Association of PD-L1 Expression with EGFR Mutation and ALK Fusion in NSCLC

As with *KRAS*-mutant-NSCLC, oncogenic Ras/Raf/MEK/ERK signaling upregulates PD-L1 expression in NSCLC with *EGFR* mutation and *ALK* fusion [74,75]. Chen et al. reported that EGFR activation, such as EGF stimulation, exon19 deletion, and exon21 L858R-mutation, upregulates PD-L1 expression through ERK/Jun phosphorylation, but not the phosphorylation of the AKT/S6 pathway [76]. In addition, IL-6/Janus Kinase (JAK)/STAT3 signaling also induces PD-L1 expression in *EGFR*-mutant NSCLC [77], whereas the PI3K-AKT pathway is involved in PD-L1 upregulation in NSCLC with *ALK* fusion [75] (Figure 1). Furthermore, several molecular mechanisms of resistance to EGFR tyrosine kinase inhibitors (EGFR-TKIs), such as hepatocyte growth factor (HGF), *c-MET* amplification, and *EGFR-T790M* mutation, were associated with upregulation of PD-L1 expression in *EGFR*-mutant NSCLC. HGF and *c-MET* amplification increase PD-L1 expression by activation of phosphoinositide 3-kinase (PI3K)/Akt, mitogen-activated protein kinase 1 (MAPK), and activator protein 1 (AP-1), whereas *EGFR-T790M* mutation increases PD-L1 expression through NF-kappa B signaling pathways in addition to signaling through PI3K/Akt/MAPK [78]. These results indicate the types of resistance mechanisms to EGFR-TKIs that promote the immune escape of tumor cells through different molecular mechanisms (Figure 1). Based on these findings, several studies showed that PD-L1 expression levels as measured by immunohistochemistry (IHC) are relatively higher in advanced NSCLC with *EGFR* mutation or *ALK* fusion compared with that of NSCLC with wild-type *EGFR* and *ALK*, though the results of some studies were inconsistent [79,80,81].

The previous studies indicate that NSCLC with *EGFR* mutation or *ALK* fusion does not respond well to PD-1/PD-L1 monotherapy compared with *EGFR-* and *ALK*-wild-type NSCLC [25,27,28,82]. These results are consistent with the findings of a lack of T-cell infiltration and low TMB in NSCLC with *EGFR* mutation or *ALK* fusion [82,83]. A pool analysis of four randomized control trials including CheckMate-057, KEYNOTE-010, OAK, and POPLAR showed that the PFS of the patients with *EGFR* mutation treated with PD-1/PD-L1 inhibitors was shorter compared with those of the patients treated with docetaxel (HR, 1.44, 95% CI (1.05–1.98); *p* = 0.02) [83]. ATLANTIC, an open-label phase 2 study, demonstrated the potential efficacy of an anti-PD-L1 monoclonal antibody, durvalumab, for third-line or later-line treatment of advanced NSCLC with *EGFR* mutation or *ALK* fusion [84]. In the present study, PD-L1 expression in the tumor was associated with PFS and objective response, indicating that ICIs should not be thoroughly excluded from candidate therapeutic strategies for NSCLC patients with *EGFR* mutation or *ALK* fusion, especially in cases with high PD-L1 expression.

### 3.4. PD-L1 Expression in NSCLC with Other Oncogenic Driver Mutations

The clinical efficacy of ICI treatment in NSCLC with *B-Raf Proto-Oncogene* (*BRAF*) mutation appears similar to that in unselected NSCLC, indicating that patients with *BRAF*-mutant NSCLC benefit more from ICI therapy than patients with NSCLC harboring an *EGFR* mutation or *ALK* fusion [85]. Zhang et al. reported that there were no significant differences in PD-L1 expression between NSCLC with *BRAF* mutation and wild-type, whereas *BRAF* mutation was associated with higher TMB compared with *BRAF* wild-type [86]. Similarly, PFS in patients with *BRAF*-mutant NSCLC treated with ICIs was approximately 10 months, which was significantly longer compared with patients harboring an *EGFR* mutation or *ALK* fusion [87]. Furthermore, there was no significant correlation between PD-L1 expression and clinical response to ICIs in *BRAF*-mutant NSCLC [85,88]. Between NSCLC with *BRAF V600E* mutation and *non-V600E* alterations, PFS and overall response rate were not significantly different, although NSCLC with *BRAF V600E* mutation exhibited lower TMB compared with those harboring *non-V600E* alterations [85] (Figure 1).

In addition to *EGFR* mutation, a multi-omics analysis identified that activating mutations in receptor tyrosine kinases (RTK) genes, such as c-MET mutation or amplification, FGFR1 amplification, human EGFR 2 (HER2) point mutation, and insulin-like growth factor 1 receptor (IGF1R) amplification, are associated with primary resistance to ICIs regardless of PD-L1 expression and TMB [21,89]. The activation of Ras/Raf/MEK/ERK signaling also upregulates PD-L1 expression in NSCLC with activated alterations of RTK genes, although IHC of PD-L1 for these genes alterations was not well studied because of the low frequency. Consistent with the results of the multi-omics analysis, V Negrao et al. reported the efficacy of ICIs in NSCLC with these low frequency driver alterations and PFS in patients with NSCLC with RTK genes alterations were relatively shorter at approximately 1.8–3.7 months [87]. In contrast, other driver gene mutations, such as JAK1/2 and AT-rich interactive domain-containing protein 1A (ARID1A) mutations, were reported to be associated with T-cell infiltration and favorable response to ICI treatment with high expression of tumor antigens as well as co-occurring *KRAS* mutations and TP53 inactivation [21,90]. These driver mutations may be useful predictive markers for PD-1/PD-L1 inhibitor response and may be useful for stratifying patients for ICI regimens.

## 4. Current Clinical Questions and Future Perspectives

The clinical development of PD-1/PD-L1 inhibitor-containing regimens has provided advanced NSCLC patients with treatment options, especially with respect to the expected toxicity profile of each regimen. However, several clinical questions regarding the efficacy of these regimens remain and additional clinical studies are ongoing.

(1) Is monotherapy with PD-1/PD-L1 inhibitors the best choice in patients with PD-L1 positive expression?

Currently, monotherapy with PD-1/PD-L1 inhibitors or immune check point inhibitors in combination with platinum-based chemotherapy may be selected. It is unclear whether the addition of platinum-based chemotherapy to PD-1/PD-L1 inhibitors is beneficial in patients with strong PD-L1 expression. NSCLC with an *ARID1A* alteration or with combined *KRAS* mutation and TP53 inactivation showed good responses to ICIs, even to monotherapy with PD-1/PD-L1 inhibitors [21,23,24,90]. To address this question, a phase III study comparing CBDCA/PEM/pembrolizumab to pembrolizumab in patients with a PD-L1 TPS ≥ 50% is ongoing [91].

(2) Are PD-1/PD-L1 inhibitors or PD-1/PD-L1 inhibitors plus CTLA-4 inhibitors a better combination with platinum-based chemotherapy in patients with NSCLC?

As mentioned earlier, quadruple regimens containing PD-1/PD-L1 inhibitors plus CTLA-4 inhibitors have become a standard treatment option, which leads us to another clinical question: Are quadruple regimens better than triplet regimen with PD-1/PD-L1 inhibitors combined with platinum-based chemotherapy? POSEIDON was a three-arm study consisting of durvalumab plus tremelimumab combined with platinum-based chemotherapy versus durvalumab combined with platinum-based chemotherapy, and platinum-based chemotherapy as the control arm. The median OS of the three regimens was 14.0 months, 13.0 months, and 11.7 months, respectively. However, the study was statistically designed to compare durvalumab plus tremelimumab in combination with platinum-based chemotherapy with the control arm (HR of OS: 0.77) and to compare durvalumab combined with platinum-based chemotherapy with the control arm (HR of OS: 0.86). Thus, direct comparison data between PD-1/PD-L1 inhibitor combinations with platinum-based chemotherapy and PD-1/PD-L1 inhibitors plus CTLA-4 inhibitors in combination with platinum-based chemotherapy are needed.

(3) Are PD-1/PD-L1 inhibitors plus platinum-based chemotherapy or PD-1/PD-L1 inhibitors plus CTLA4 inhibitors beneficial in NSCLC patients harboring sensitizing EGFR mutations?

IMpower150 demonstrated the potential benefits of adding PD-1/PD-L1 inhibitors to platinum-based chemotherapy in NSCLC patients with a sensitizing EGFR mutation. However, it remains unclear whether such patients benefited from a PD-1/PD-L1 inhibitor-containing regimen. A phase III study comparing nivolumab plus pemetrexed/platinum or nivolumab/ipilimumab to pemetrexed plus platinum in NSCLC patients with EGFR mutations following failure with EGFR tyrosine kinase inhibitor therapy is ongoing (NCT02864251). Furthermore, an ongoing phase III study comparing atezolizumab plus CBDCA/PEM/BEV to CBDCA/PEM/BEV is designed to include patients with an EGFR mutation or ALK fusion who showed treatment failure with an approved tyrosine kinase inhibitor (JapicCTI-194565) [92]. These studies would resolve the questions of the role of PD-1/PD-L1 inhibitors in NSCLC patients with a sensitizing EGFR mutation.

(4) Is reiteration of PD-1/PD-L1 inhibitors effective to a subset of the NSCLC population?

The PACIFIC trial demonstrated clinical efficacy of durvalumab monotherapy for patients with locally advanced and unresectable NSCLC after concurrent platinum-based chemoradiotherapy [93]. However, the clinical benefits of the reiteration of PD-1/PD-L1 regimens for patients with recurrent disease after durvalumab are unknown. Regarding the reiteration of PD-1/PD-L1 inhibitors in advanced NSCLC, there are a few case studies to address the efficacy [94,95,96]; however, most of these cases included other therapies before, after, and between PD-1/PD-L1 inhibitor treatment, thus additional effects of cytotoxic chemotherapy or abscopal effects of radiotherapy may impact the analysis. Furthermore, there is the possibility of severe adverse events caused by reiteration of PD-1/PD-L1 inhibitor therapy. Therefore, further clinical trials that include patients with recurrent NSCLC after durvalumab are needed.

## 5. Conclusions

In summary, there are now multiple treatment options that contain PD-1/PD-L1 inhibitor regimens for chemotherapy naïve advanced NSCLC patients. The optimal use of these treatment options is one of the most important issues in the area of immunotherapy. Several head-to-head trials to investigate which options are more effective for patients with NSCLC. PD-L1 expression status may be used to stratify patients for these trials, although it is known as a week indicator to predict clinical outcome. In parallel to the development of therapeutic strategies using ICIs, recent molecular approaches have begun to elucidate the relationship between key driver mutations and distinct immune phenotypes in NSCLC. A better understanding of these relationships will help in the selection of responders for ICI therapy and to design future clinical trials for precision medicine. In particular, new therapeutic developments for immune resistant phenotypes, such as NSCLC with LKB1 and/or KEAP1 inactivation, are urgently needed to improve the extremely poor prognosis. Combining clinical trial results with molecular biological findings will drive the selection of suitable ICI therapies for patients based on PD-L1 expression status and key driver mutations.

## Figures and Tables

**Table 1:** Pivotal evidence of PD-1/PD-L1 inhibitors regimen showing clinical benefit to platinum-based chemotherapy for untreated NSCLC.

Clinical Study	Patient	Experimental Arm	Control Arm	PFS	OS
KEYNOTE-024	NSCLC, PD-L1 TPS ≥ 50%	Pembrolizumab	Platinum-based chemotherapy	HR 0.50 (95% CI, 0.37–0.68)	HR 0.60 (95% CI, 0.41–0.89)
KEYNOTE-042	NSCLC, PD-L1 TPS ≥ 1%	Pembrolizumab	Platinum-based chemotherapy	HR 1.07 (95% CI, 0.94–1.21)	HR 0.81 (95% CI, 0.71–0.93)
IMpower110	NSCLC, PD-L1 TC3 or IC3	Atezolizumab	Platinum-based chemotherapy	HR 0.63 (95% CI, 0.45–0.88)	HR 0.59 (95% CI, 0.40–0.89)
KEYNOTE-189	Non-Sq NSCLC	Pembrolizumab + CDDP/CBDCA + PEM	CDDP/CBDCA+PEM	HR 0.52 (95% CI, 0.43–0.64)	HR 0.49 (95% CI, 0.38–0.64)
KEYNOTE-407	Sq NSCLC	Pembrolizumab + CBDCA + nabPTX/PTX	CBDCA+nabPTX/PTX	HR 0.56 (95% CI, 0.45–0.70)	HR 0.64 (95% CI, 0.49–0.85)
IMpower130	Non-Sq NSCLC	Atezolizumab + CBDCA + nabPTX	CBDCA+nabPTX	HR 0.64 (95% CI, 0.54–0.77)	HR 0.79 (95% CI, 0.64–0.98)
IMpower132	Non-Sq NSCLC	Atezolizumab + CDDP/CBDCA + PEM	CDDP/CBDCA+PEM	HR 0.60 (95% CI, 0.49–0.72)	HR 0.86 (95% CI, 0.71–1.06)
IMpower150	Non-Sq NSCLC	Atezolizumab + CBDCA + PTX + BEV	CBDCA+PTX+BEV	HR 0.62 (95% CI, 0.52–0.74)	HR 0.78 (95% CI, 0.64–0.96)
ONO-4538–52/TASUKI-52	Non-Sq NSCLC	Nivolumab + CBDCA+PTX + BEV	CBDCA+PTX+BEV	HR 0.56 (95% CI, 0.43–0.71)	HR 0.85 (95% CI, 0.63–1.14)
POSEIDON	NSCLC	Durvalumab + Platinum-based chemotherapy	Platinum-based chemotherapy	HR 0.74 (95% CI, 0.62–0.89)	HR 0.86 (95% CI, 0.72–1.02)
CheckMate 227	NSCLC PD-L1 level ≥ 1%	Nivolumab + Ipilimumab	Platinum-based chemotherapy	HR 0.82 (95%CI, 0.69–0.97)	HR 0.79 (97.72% CI, 0.65–0.96)
CheckMate 9LA	NSCLC	Nivolumab + Ipilimumab + Platinum based chemotherapy	Platinum-based chemotherapy	HR 0.70 (97.48%CI, 0.57–0.86)	HR 0.69 (96.71% CI, 0.55–0.87)
POSEIDON	NSCLC	Durvalumab + Tremelimumab + Platinum-based chemotherapy	Platinum-based chemotherapy	HR 0.72 (95% CI, 0.60–0.86)	HR 0.77 (95% CI, 0.65–0.92)

TPS, Tumor proportion score; CDDP, cisplatin; CBDCA, carboplatin; PEM, pemetrexed; nab-PTX, nanoparticle albumin bound-Paclitaxel; PTX, Paclitaxel; BEV, bevacizumab.

## Data Availability

Not applicable.
